# 4-{2-[4-(Dimethyl­amino)­phen­yl]ethen­yl}-1-methyl­pyridinium 3,5-dicarb­oxy­benzene­sulfonate methanol monosolvate

**DOI:** 10.1107/S1600536811054419

**Published:** 2012-01-07

**Authors:** Liang Li, Huijuan Cui, Zhou Yang, Xutang Tao, Huai Yang

**Affiliations:** a Department of Materials Physics and Chemistry, School of Materials Science and Engineering, University of Science and Technology Beijing, Beijing 100083, People’s Republic of China; b State Key Laboratory of Crystal Materials, Shandong University, Jinan, 250100, People’s Republic of China

## Abstract

In the crystal structure of the title solvated salt, C_16_H_19_N_2_^+^·C_8_H_5_O_7_S^−^·CH_3_OH, the anions and the methanol solvent mol­ecules are linked by O—H⋯O hydrogen bonds. The cations and anions are packed as alternate layers parallel to (11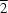). The crystal structure is further stabilized by a π–π inter­action between the pyridinium and benzene rings of the cations, with a centroid–centroid distance of 3.5492 (4) Å.

## Related literature

The title compound was synthesized as part of our continuing research on the nonlinear optical properties of DAS (4-*N*,*N*-dimethyl­amino-4′-*N*′-methyl­stilbazolium) derivatives. For the synthesis, see: Okada *et al.* (1990 ▶). For background to non-linear optical materials, see: Bosshard *et al.* (1995 ▶); Nalwa & Miyata (1997 ▶); Yang, Mutter *et al.* (2007 ▶); Ruiz *et al.* (2006 ▶). For the effects of different substituents of benzene sulfonate on its non-linear optical properties, see: Okada *et al.* (2003 ▶); Yang, Wörle *et al.* (2007 ▶); Ogawa *et al.* (2008 ▶), Yang *et al.* (2005 ▶). For standard bond-lengths, see: Allen *et al.* (1987 ▶).
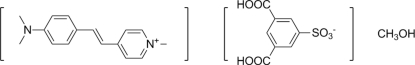


## Experimental

### 

#### Crystal data

C_16_H_19_N_2_^+^·C_8_H_5_O_7_S^−^·CH_4_O
*M* *_r_* = 516.55
Triclinic, 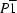

*a* = 7.5277 (12) Å
*b* = 10.5517 (17) Å
*c* = 16.467 (3) Å
α = 106.750 (7)°
β = 97.504 (8)°
γ = 100.273 (8)°
*V* = 1209.2 (3) Å^3^
*Z* = 2
Mo *K*α radiation
μ = 0.19 mm^−1^
*T* = 173 K
0.45 × 0.31 × 0.22 mm

#### Data collection

Rigaku Saturn724+ CCD diffractometer
Absorption correction: multi-scan (*CrystalClear*; Rigaku, 2008 ▶) *T*_min_ = 0.629, *T*_max_ = 1.000
15622 measured reflections
5523 independent reflections
5192 reflections with *I* > 2σ(*I*)
*R*_int_ = 0.037

#### Refinement

*R*[*F* ^2^ > 2σ(*F* ^2^)] = 0.048
*wR*(*F* ^2^) = 0.116
*S* = 1.13
5523 reflections
332 parameters
H-atom parameters constrained
Δρ_max_ = 0.33 e Å^−3^
Δρ_min_ = −0.33 e Å^−3^

### 

Data collection: *CrystalClear* (Rigaku, 2008 ▶); cell refinement: *CrystalClear*; data reduction: *CrystalClear*; program(s) used to solve structure: *SHELXS97* (Sheldrick, 2008 ▶); program(s) used to refine structure: *SHELXL97* (Sheldrick, 2008 ▶); molecular graphics: *Mercury* (Macrae *et al.*, 2006 ▶); software used to prepare material for publication: *SHELXL97*.

## Supplementary Material

Crystal structure: contains datablock(s) I, global. DOI: 10.1107/S1600536811054419/ez2270sup1.cif

Structure factors: contains datablock(s) I. DOI: 10.1107/S1600536811054419/ez2270Isup2.hkl

Supplementary material file. DOI: 10.1107/S1600536811054419/ez2270Isup3.cdx

Supplementary material file. DOI: 10.1107/S1600536811054419/ez2270Isup4.cml

Additional supplementary materials:  crystallographic information; 3D view; checkCIF report

**Fig. 1.**
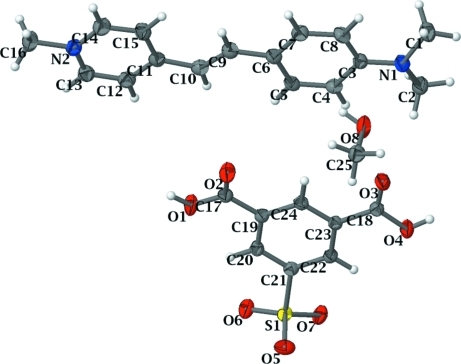
The molecular structure of the title compound showing the atom numbering scheme and 50% probability displacement ellipsoids.

Enhanced figure: interactive version of Fig. 1

## Figures and Tables

**Table 1:** Hydrogen-bond geometry (Å, °)

*D*—H⋯*A*	*D*—H	H⋯*A*	*D*⋯*A*	*D*—H⋯*A*
O1—H1⋯O6^i^	0.84	1.89	2.6796 (18)	156
O4—H4*A*⋯O8^ii^	0.84	1.79	2.6156 (18)	168
O8—H8⋯O7^iii^	0.84	1.86	2.6897 (18)	169

Symmetry codes: (i) 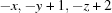; (ii) 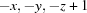; (iii) 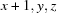.

**Fig. 2.**
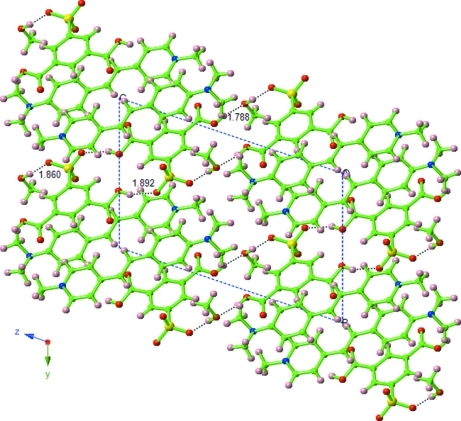
A packing diagram of the title compound showing the hydrogen bonds as dashed lines.

## supplementary crystallographic information

### Comment

During the last three decades, nonlinear optical materials have been of considerable interest for their potential applications such as frequency conversion, electro-optic modulation and THz generation (Bosshard *et al.*, 1995; Nalwa & Miyata, 1997; Yang, Mutter *et al.*, 2007). In order to create efficient NLO materials, both the molecular and bulk properties must be optimized (Yang *et al.*, 2005; Ruiz *et al.*, 2006; Yang, Wörle *et al.*, 2007). The title compound was synthesized as part of our continuing research on the nonlinear optical properties of DAS (4-N, N-dimethylamino-4'-N'-methyl-stilbazolium) derivatives.

Fig. 1 illustrates the molecular structure of the title compound together with the atomic numbering scheme. The unit cell of the title compound contains two asymmetric units, each consisting of one C_16_H_19_N_2_^+^ cation, one C_8_H_5_O_7_S^-^ anion and one methanol molecule. The bond distances and angles in both the cation and anion are in normal ranges (Allen *et al.*, 1987). In the crystal structure, atoms O4, O6, O7 and O8 of the anion are involved in O—H···O interactions. The cations and anions are stacked in a parallel manner and form alternating layers parallel to the (112) plane. The crystal structure is further stabilized by a π···π interaction between the pyridinium and C3–C8 benzene rings with a centroid–centroid distance of 3.5492 (4) Å, which combine with the intermolecular O—H···O interactions to form a three-dimensional network.

### Experimental

4-{2-[4-(Dimethylamino)phenyl]ethenyl}-1-methylpyridinium 3,5-dicarboxybenzenesulfonate was prepared by the metathesization of 4-N,N-dimethylamino-4'-N'-methyl-stilbazolium iodide (Okada *et al.*, 1990) with the sodium salt of the 3,5-dicarboxybenzenesulfonic acid. The title compound was then recrystallized from methanol to get high purity material for crystal growth. 4-{2-[4-(Dimethylamino)phenyl]ethenyl}-1-methylpyridinium 3,5-dicarboxybenzenesulfonate: yield 72%; ^1^H-NMR (400 MHz, DMSO-d_6_): 8.67 (d, 2H, J= 6.8Hz, C_5_H_4_N), 8.39 (s, 1H, C_6_H_3_SO_3_^-^), 8.34 (d, 2H, C_6_H_3_SO_3_^-^), 8.03(d, 2H, J= 6.8Hz, C_5_H_4_N), 7.91 (d, 1H, J= 16.0Hz, CH), 7.59(d, 2H, J= 8.4Hz, C_6_H_3_SO_3_^-^), 7.17 (d, 1H, J= 16.0Hz, CH), 6.79 (d, 2H, J= 8.8Hz, C_6_H_4_), 4.16 (s, 3H, NMe), 3.01 (s, 6H, NMe_2_). C, H, N analysis calcd. for C_24_H_24_N_2_O_7_S: C 59.49, H 4.99, N 5.78; found: C 59.39, H 5.02, N 5.79. Crystals were obtained by slow cooling method from 45°C to room temperature in methanol.

### Refinement

All H atoms were located geometrically (methyl C-H = 0.98 Å, aromatic C-H = 0.95Å and O-H = 0.84 Å) and refined using a riding model, with U_iso_(H) = 1.2 or 1.5U_eq_(C).

### Crystal data

**Table:**

C_16_H_19_N_2_^+^·C_8_H_5_O_7_S^−^·CH_4_O	*Z* = 2
*M**_r_* = 516.55	*F*(000) = 544
Triclinic, *P*1	*D*_x_ = 1.419 Mg m^−^^3^
*a* = 7.5277 (12) Å	Mo *K*α radiation, λ = 0.71073 Å
*b* = 10.5517 (17) Å	Cell parameters from 4222 reflections
*c* = 16.467 (3) Å	θ = 1.3–27.5°
α = 106.750 (7)°	µ = 0.19 mm^−^^1^
β = 97.504 (8)°	*T* = 173 K
γ = 100.273 (8)°	Block, red
*V* = 1209.2 (3) Å^3^	0.45 × 0.31 × 0.22 mm

### Data collection

**Table:**

Rigaku Saturn724+ CCD diffractometer	5523 independent reflections
Radiation source: fine-focus sealed tube	5192 reflections with *I* > 2σ(*I*)
graphite	*R*_int_ = 0.037
Detector resolution: 28.5714 pixels mm^-1^	θ_max_ = 27.5°, θ_min_ = 2.8°
ω scans at fixed χ = 45°	*h* = −9→9
Absorption correction: multi-scan (*CrystalClear*; Rigaku, 2008)	*k* = −13→13
*T*_min_ = 0.629, *T*_max_ = 1.000	*l* = −21→21
15622 measured reflections	

### Refinement

**Table:**

Refinement on *F*^2^	Primary atom site location: structure-invariant direct methods
Least-squares matrix: full	Secondary atom site location: difference Fourier map
*R*[*F*^2^ > 2σ(*F*^2^)] = 0.048	Hydrogen site location: inferred from neighbouring sites
*wR*(*F*^2^) = 0.116	H-atom parameters constrained
*S* = 1.13	*w* = 1/[σ^2^(*F*_o_^2^) + (0.0477*P*)^2^ + 0.4666*P*] where *P* = (*F*_o_^2^ + 2*F*_c_^2^)/3
5523 reflections	(Δ/σ)_max_ = 0.001
332 parameters	Δρ_max_ = 0.33 e Å^−^^3^
0 restraints	Δρ_min_ = −0.33 e Å^−^^3^

### Special details

**Table:**

Geometry. All e.s.d.'s (except the e.s.d. in the dihedral angle between two l.s. planes) are estimated using the full covariance matrix. The cell e.s.d.'s are taken into account individually in the estimation of e.s.d.'s in distances, angles and torsion angles; correlations between e.s.d.'s in cell parameters are only used when they are defined by crystal symmetry. An approximate (isotropic) treatment of cell e.s.d.'s is used for estimating e.s.d.'s involving l.s. planes.
Refinement. Refinement of *F*^2^ against ALL reflections. The weighted *R*-factor *wR* and goodness of fit *S* are based on *F*^2^, conventional *R*-factors *R* are based on *F*, with *F* set to zero for negative *F*^2^. The threshold expression of *F*^2^ > σ(*F*^2^) is used only for calculating *R*-factors(gt) *etc*. and is not relevant to the choice of reflections for refinement. *R*-factors based on *F*^2^ are statistically about twice as large as those based on *F*, and *R*- factors based on ALL data will be even larger.

### Fractional atomic coordinates and isotropic or equivalent isotropic displacement parameters (Å^2^)

**Table:**

	*x*	*y*	*z*	*U*_iso_*/*U*_eq_	
S1	−0.38860 (5)	0.41490 (4)	0.77513 (3)	0.02588 (12)	
O1	0.1455 (2)	0.37615 (12)	0.99887 (8)	0.0390 (3)	
H1	0.2036	0.3798	1.0470	0.059*	
O2	0.1780 (2)	0.16194 (13)	0.96041 (8)	0.0420 (3)	
O3	−0.09373 (18)	−0.12371 (12)	0.64695 (8)	0.0350 (3)	
O4	−0.28863 (18)	−0.04200 (13)	0.57227 (8)	0.0346 (3)	
H4A	−0.2926	−0.1141	0.5327	0.052*	
O5	−0.55084 (17)	0.36035 (13)	0.80299 (9)	0.0374 (3)	
O6	−0.28648 (18)	0.54879 (12)	0.83184 (8)	0.0347 (3)	
O7	−0.42469 (17)	0.41121 (12)	0.68482 (8)	0.0332 (3)	
O8	0.2880 (2)	0.24475 (14)	0.56382 (8)	0.0436 (4)	
H8	0.3854	0.2890	0.5993	0.065*	
N1	0.3505 (2)	−0.25772 (15)	0.60065 (10)	0.0338 (3)	
N2	0.9237 (2)	0.38403 (14)	1.25311 (10)	0.0313 (3)	
C1	0.4220 (3)	−0.37550 (19)	0.56160 (13)	0.0406 (4)	
H1C	0.3964	−0.4424	0.5918	0.061*	
H1A	0.5552	−0.3472	0.5662	0.061*	
H1B	0.3626	−0.4162	0.5006	0.061*	
C2	0.2606 (3)	−0.1979 (2)	0.54209 (12)	0.0401 (4)	
H2B	0.3443	−0.1152	0.5424	0.060*	
H2A	0.1488	−0.1752	0.5610	0.060*	
H2C	0.2280	−0.2631	0.4835	0.060*	
C3	0.4215 (2)	−0.18111 (16)	0.68546 (11)	0.0261 (3)	
C4	0.3830 (2)	−0.05246 (17)	0.72159 (11)	0.0281 (3)	
H4	0.3044	−0.0190	0.6870	0.034*	
C5	0.4577 (2)	0.02533 (16)	0.80627 (11)	0.0271 (3)	
H5	0.4301	0.1116	0.8288	0.033*	
C6	0.5736 (2)	−0.02072 (16)	0.85963 (11)	0.0271 (3)	
C7	0.6059 (2)	−0.15028 (17)	0.82433 (11)	0.0297 (4)	
H7	0.6799	−0.1853	0.8599	0.036*	
C8	0.5340 (2)	−0.22835 (16)	0.73985 (11)	0.0297 (4)	
H8A	0.5606	−0.3151	0.7180	0.036*	
C9	0.6622 (2)	0.05654 (17)	0.94810 (11)	0.0290 (4)	
H9	0.7296	0.0107	0.9785	0.035*	
C10	0.6608 (2)	0.18460 (17)	0.99228 (11)	0.0296 (4)	
H10	0.5937	0.2334	0.9642	0.036*	
C11	0.7569 (2)	0.25224 (17)	1.08083 (11)	0.0282 (3)	
C12	0.7411 (2)	0.38370 (18)	1.12496 (12)	0.0325 (4)	
H12	0.6721	0.4299	1.0956	0.039*	
C13	0.8239 (2)	0.44591 (18)	1.20961 (12)	0.0333 (4)	
H13	0.8107	0.5347	1.2384	0.040*	
C14	0.9459 (3)	0.25904 (18)	1.21244 (12)	0.0351 (4)	
H14	1.0186	0.2166	1.2432	0.042*	
C15	0.8659 (2)	0.19230 (17)	1.12795 (12)	0.0333 (4)	
H15	0.8840	0.1044	1.1006	0.040*	
C16	1.0116 (3)	0.4516 (2)	1.34462 (12)	0.0421 (5)	
H16B	0.9368	0.5113	1.3731	0.063*	
H16C	1.1346	0.5056	1.3485	0.063*	
H16A	1.0221	0.3828	1.3732	0.063*	
C17	0.1147 (2)	0.25092 (16)	0.94444 (11)	0.0280 (3)	
C18	−0.1751 (2)	−0.03581 (16)	0.64270 (10)	0.0256 (3)	
C19	−0.0110 (2)	0.23178 (16)	0.86173 (10)	0.0242 (3)	
C20	−0.1150 (2)	0.32637 (15)	0.85514 (10)	0.0242 (3)	
H20	−0.1007	0.4069	0.9023	0.029*	
C21	−0.2395 (2)	0.30264 (15)	0.77944 (10)	0.0231 (3)	
C22	−0.2594 (2)	0.18634 (15)	0.70939 (10)	0.0241 (3)	
H22	−0.3434	0.1714	0.6574	0.029*	
C23	−0.1550 (2)	0.09165 (15)	0.71595 (10)	0.0236 (3)	
C24	−0.0322 (2)	0.11394 (16)	0.79225 (10)	0.0248 (3)	
H24	0.0373	0.0487	0.7970	0.030*	
C25	0.1596 (3)	0.19091 (19)	0.60747 (12)	0.0368 (4)	
H25A	0.1064	0.0948	0.5753	0.055*	
H25C	0.0616	0.2410	0.6118	0.055*	
H25B	0.2218	0.1996	0.6656	0.055*	

### Atomic displacement parameters (Å^2^)

**Table:**

	*U*^11^	*U*^22^	*U*^33^	*U*^12^	*U*^13^	*U*^23^
S1	0.0280 (2)	0.02105 (19)	0.0269 (2)	0.00778 (15)	−0.00144 (16)	0.00678 (15)
O1	0.0533 (8)	0.0281 (6)	0.0274 (6)	0.0160 (6)	−0.0121 (6)	0.0009 (5)
O2	0.0555 (8)	0.0343 (7)	0.0324 (7)	0.0236 (6)	−0.0088 (6)	0.0042 (5)
O3	0.0403 (7)	0.0317 (6)	0.0295 (6)	0.0159 (5)	0.0021 (5)	0.0017 (5)
O4	0.0414 (7)	0.0339 (7)	0.0227 (6)	0.0112 (6)	−0.0017 (5)	0.0020 (5)
O5	0.0325 (7)	0.0365 (7)	0.0499 (8)	0.0159 (5)	0.0121 (6)	0.0171 (6)
O6	0.0427 (7)	0.0219 (6)	0.0335 (7)	0.0094 (5)	−0.0085 (6)	0.0055 (5)
O7	0.0386 (7)	0.0299 (6)	0.0284 (6)	0.0083 (5)	−0.0059 (5)	0.0101 (5)
O8	0.0445 (8)	0.0437 (8)	0.0262 (6)	−0.0102 (6)	0.0031 (6)	0.0001 (6)
N1	0.0385 (8)	0.0296 (7)	0.0305 (8)	0.0129 (6)	0.0022 (6)	0.0042 (6)
N2	0.0292 (7)	0.0294 (7)	0.0306 (8)	0.0019 (6)	0.0038 (6)	0.0064 (6)
C1	0.0514 (12)	0.0325 (9)	0.0338 (10)	0.0152 (8)	0.0048 (9)	0.0027 (8)
C2	0.0436 (11)	0.0466 (11)	0.0281 (9)	0.0186 (9)	0.0003 (8)	0.0065 (8)
C3	0.0260 (8)	0.0239 (8)	0.0274 (8)	0.0048 (6)	0.0058 (7)	0.0069 (6)
C4	0.0286 (8)	0.0272 (8)	0.0318 (9)	0.0102 (7)	0.0061 (7)	0.0123 (7)
C5	0.0296 (8)	0.0230 (7)	0.0304 (8)	0.0079 (6)	0.0096 (7)	0.0081 (6)
C6	0.0276 (8)	0.0269 (8)	0.0280 (8)	0.0048 (6)	0.0079 (7)	0.0106 (6)
C7	0.0327 (9)	0.0301 (8)	0.0303 (9)	0.0107 (7)	0.0057 (7)	0.0135 (7)
C8	0.0356 (9)	0.0229 (8)	0.0337 (9)	0.0106 (7)	0.0096 (7)	0.0101 (7)
C9	0.0304 (9)	0.0297 (8)	0.0295 (9)	0.0072 (7)	0.0074 (7)	0.0126 (7)
C10	0.0294 (9)	0.0298 (8)	0.0323 (9)	0.0080 (7)	0.0067 (7)	0.0130 (7)
C11	0.0261 (8)	0.0284 (8)	0.0300 (9)	0.0032 (6)	0.0085 (7)	0.0095 (7)
C12	0.0320 (9)	0.0296 (8)	0.0368 (9)	0.0092 (7)	0.0046 (7)	0.0118 (7)
C13	0.0320 (9)	0.0267 (8)	0.0389 (10)	0.0072 (7)	0.0067 (8)	0.0066 (7)
C14	0.0360 (10)	0.0310 (9)	0.0374 (10)	0.0089 (7)	0.0017 (8)	0.0114 (7)
C15	0.0385 (10)	0.0257 (8)	0.0345 (9)	0.0098 (7)	0.0058 (8)	0.0071 (7)
C16	0.0416 (11)	0.0423 (11)	0.0315 (10)	0.0009 (9)	0.0004 (8)	0.0034 (8)
C17	0.0295 (8)	0.0271 (8)	0.0267 (8)	0.0116 (7)	0.0024 (7)	0.0057 (6)
C18	0.0243 (8)	0.0284 (8)	0.0229 (8)	0.0049 (6)	0.0049 (6)	0.0070 (6)
C19	0.0246 (8)	0.0247 (7)	0.0233 (8)	0.0066 (6)	0.0040 (6)	0.0075 (6)
C20	0.0266 (8)	0.0209 (7)	0.0240 (8)	0.0051 (6)	0.0039 (6)	0.0062 (6)
C21	0.0229 (7)	0.0216 (7)	0.0261 (8)	0.0057 (6)	0.0044 (6)	0.0094 (6)
C22	0.0238 (8)	0.0256 (8)	0.0226 (7)	0.0050 (6)	0.0030 (6)	0.0082 (6)
C23	0.0237 (7)	0.0238 (7)	0.0230 (8)	0.0051 (6)	0.0061 (6)	0.0067 (6)
C24	0.0261 (8)	0.0250 (7)	0.0251 (8)	0.0096 (6)	0.0056 (6)	0.0080 (6)
C25	0.0352 (10)	0.0354 (9)	0.0347 (10)	0.0039 (8)	0.0056 (8)	0.0062 (8)

### Geometric parameters (Å, °)

**Table:**

S1—O5	1.4427 (13)	C7—C8	1.375 (2)
S1—O6	1.4572 (12)	C7—H7	0.9500
S1—O7	1.4643 (13)	C8—H8A	0.9500
S1—C21	1.7801 (16)	C9—C10	1.340 (2)
O1—C17	1.325 (2)	C9—H9	0.9500
O1—H1	0.8400	C10—C11	1.449 (2)
O2—C17	1.203 (2)	C10—H10	0.9500
O3—C18	1.212 (2)	C11—C12	1.402 (2)
O4—C18	1.326 (2)	C11—C15	1.406 (2)
O4—H4A	0.8400	C12—C13	1.364 (3)
O8—C25	1.412 (2)	C12—H12	0.9500
O8—H8	0.8400	C13—H13	0.9500
N1—C3	1.375 (2)	C14—C15	1.364 (3)
N1—C1	1.455 (2)	C14—H14	0.9500
N1—C2	1.456 (2)	C15—H15	0.9500
N2—C13	1.345 (2)	C16—H16B	0.9800
N2—C14	1.347 (2)	C16—H16C	0.9800
N2—C16	1.473 (2)	C16—H16A	0.9800
C1—H1C	0.9800	C17—C19	1.493 (2)
C1—H1A	0.9800	C18—C23	1.494 (2)
C1—H1B	0.9800	C19—C20	1.393 (2)
C2—H2B	0.9800	C19—C24	1.394 (2)
C2—H2A	0.9800	C20—C21	1.388 (2)
C2—H2C	0.9800	C20—H20	0.9500
C3—C8	1.406 (2)	C21—C22	1.390 (2)
C3—C4	1.414 (2)	C22—C23	1.396 (2)
C4—C5	1.380 (2)	C22—H22	0.9500
C4—H4	0.9500	C23—C24	1.392 (2)
C5—C6	1.402 (2)	C24—H24	0.9500
C5—H5	0.9500	C25—H25A	0.9800
C6—C7	1.401 (2)	C25—H25C	0.9800
C6—C9	1.449 (2)	C25—H25B	0.9800
			
O5—S1—O6	114.32 (8)	C12—C11—C15	116.21 (16)
O5—S1—O7	113.04 (8)	C12—C11—C10	120.18 (16)
O6—S1—O7	111.46 (7)	C15—C11—C10	123.60 (16)
O5—S1—C21	105.50 (7)	C13—C12—C11	120.71 (17)
O6—S1—C21	106.09 (7)	C13—C12—H12	119.6
O7—S1—C21	105.58 (7)	C11—C12—H12	119.6
C17—O1—H1	109.5	N2—C13—C12	121.34 (16)
C18—O4—H4A	109.5	N2—C13—H13	119.3
C25—O8—H8	109.5	C12—C13—H13	119.3
C3—N1—C1	119.25 (15)	N2—C14—C15	121.23 (17)
C3—N1—C2	119.96 (15)	N2—C14—H14	119.4
C1—N1—C2	116.88 (15)	C15—C14—H14	119.4
C13—N2—C14	119.76 (16)	C14—C15—C11	120.71 (16)
C13—N2—C16	120.98 (16)	C14—C15—H15	119.6
C14—N2—C16	119.25 (16)	C11—C15—H15	119.6
N1—C1—H1C	109.5	N2—C16—H16B	109.5
N1—C1—H1A	109.5	N2—C16—H16C	109.5
H1C—C1—H1A	109.5	H16B—C16—H16C	109.5
N1—C1—H1B	109.5	N2—C16—H16A	109.5
H1C—C1—H1B	109.5	H16B—C16—H16A	109.5
H1A—C1—H1B	109.5	H16C—C16—H16A	109.5
N1—C2—H2B	109.5	O2—C17—O1	123.90 (16)
N1—C2—H2A	109.5	O2—C17—C19	123.72 (15)
H2B—C2—H2A	109.5	O1—C17—C19	112.37 (13)
N1—C2—H2C	109.5	O3—C18—O4	123.48 (15)
H2B—C2—H2C	109.5	O3—C18—C23	123.32 (15)
H2A—C2—H2C	109.5	O4—C18—C23	113.19 (14)
N1—C3—C8	121.18 (15)	C20—C19—C24	119.86 (15)
N1—C3—C4	121.36 (15)	C20—C19—C17	120.65 (14)
C8—C3—C4	117.45 (15)	C24—C19—C17	119.37 (14)
C5—C4—C3	121.16 (15)	C21—C20—C19	119.82 (14)
C5—C4—H4	119.4	C21—C20—H20	120.1
C3—C4—H4	119.4	C19—C20—H20	120.1
C4—C5—C6	121.17 (15)	C20—C21—C22	120.65 (14)
C4—C5—H5	119.4	C20—C21—S1	119.61 (12)
C6—C5—H5	119.4	C22—C21—S1	119.54 (12)
C7—C6—C5	117.35 (15)	C21—C22—C23	119.52 (15)
C7—C6—C9	118.11 (15)	C21—C22—H22	120.2
C5—C6—C9	124.54 (15)	C23—C22—H22	120.2
C8—C7—C6	122.09 (16)	C24—C23—C22	120.01 (14)
C8—C7—H7	119.0	C24—C23—C18	118.89 (14)
C6—C7—H7	119.0	C22—C23—C18	121.09 (14)
C7—C8—C3	120.72 (15)	C23—C24—C19	120.12 (15)
C7—C8—H8A	119.6	C23—C24—H24	119.9
C3—C8—H8A	119.6	C19—C24—H24	119.9
C10—C9—C6	128.01 (16)	O8—C25—H25A	109.5
C10—C9—H9	116.0	O8—C25—H25C	109.5
C6—C9—H9	116.0	H25A—C25—H25C	109.5
C9—C10—C11	123.11 (16)	O8—C25—H25B	109.5
C9—C10—H10	118.4	H25A—C25—H25B	109.5
C11—C10—H10	118.4	H25C—C25—H25B	109.5
			
C1—N1—C3—C8	−12.7 (3)	C10—C11—C15—C14	−176.93 (16)
C2—N1—C3—C8	−169.80 (16)	O2—C17—C19—C20	163.92 (17)
C1—N1—C3—C4	167.82 (16)	O1—C17—C19—C20	−14.8 (2)
C2—N1—C3—C4	10.8 (3)	O2—C17—C19—C24	−12.2 (3)
N1—C3—C4—C5	−178.64 (15)	O1—C17—C19—C24	169.03 (15)
C8—C3—C4—C5	1.9 (2)	C24—C19—C20—C21	0.2 (2)
C3—C4—C5—C6	−0.3 (3)	C17—C19—C20—C21	−175.99 (15)
C4—C5—C6—C7	−1.9 (2)	C19—C20—C21—C22	−1.2 (2)
C4—C5—C6—C9	177.82 (16)	C19—C20—C21—S1	173.64 (12)
C5—C6—C7—C8	2.5 (2)	O5—S1—C21—C20	−89.52 (14)
C9—C6—C7—C8	−177.17 (15)	O6—S1—C21—C20	32.14 (15)
C6—C7—C8—C3	−1.0 (3)	O7—S1—C21—C20	150.54 (13)
N1—C3—C8—C7	179.29 (16)	O5—S1—C21—C22	85.32 (14)
C4—C3—C8—C7	−1.2 (2)	O6—S1—C21—C22	−153.02 (13)
C7—C6—C9—C10	174.95 (17)	O7—S1—C21—C22	−34.61 (14)
C5—C6—C9—C10	−4.7 (3)	C20—C21—C22—C23	1.0 (2)
C6—C9—C10—C11	−179.27 (15)	S1—C21—C22—C23	−173.75 (12)
C9—C10—C11—C12	−176.31 (16)	C21—C22—C23—C24	0.0 (2)
C9—C10—C11—C15	2.3 (3)	C21—C22—C23—C18	178.81 (14)
C15—C11—C12—C13	−1.9 (3)	O3—C18—C23—C24	2.3 (2)
C10—C11—C12—C13	176.88 (16)	O4—C18—C23—C24	−176.68 (14)
C14—N2—C13—C12	1.1 (3)	O3—C18—C23—C22	−176.44 (16)
C16—N2—C13—C12	−179.62 (17)	O4—C18—C23—C22	4.5 (2)
C11—C12—C13—N2	0.5 (3)	C22—C23—C24—C19	−1.0 (2)
C13—N2—C14—C15	−1.2 (3)	C18—C23—C24—C19	−179.82 (14)
C16—N2—C14—C15	179.51 (17)	C20—C19—C24—C23	0.9 (2)
N2—C14—C15—C11	−0.3 (3)	C17—C19—C24—C23	177.13 (15)
C12—C11—C15—C14	1.8 (3)		

### Hydrogen-bond geometry (Å, °)

**Table:**

*D*—H···*A*	*D*—H	H···*A*	*D*···*A*	*D*—H···*A*
O1—H1···O6^i^	0.84	1.89	2.6796 (18)	156.
O4—H4A···O8^ii^	0.84	1.79	2.6156 (18)	168.
O8—H8···O7^iii^	0.84	1.86	2.6897 (18)	169.

Symmetry codes: (i) −*x*, −*y*+1, −*z*+2; (ii) −*x*, −*y*, −*z*+1; (iii) *x*+1, *y*, *z*.
